# Head-to-Head Comparison of ^68^Ga-DOTA-TATE and ^68^Ga-DOTA-JR11 PET/CT in Patients With Tumor-Induced Osteomalacia: A Prospective Study

**DOI:** 10.3389/fonc.2022.811209

**Published:** 2022-02-24

**Authors:** Guozhu Hou, Yuwei Zhang, Yu Liu, Peipei Wang, Weibo Xia, Xiaoping Xing, Li Huo, Fang Li, Hongli Jing

**Affiliations:** ^1^ Department of Nuclear Medicine, State Key Laboratory of Complex Severe and Rare Diseases, Peking Union Medical College Hospital Chinese Academy of Medical Sciences and Peking Union Medical College, Beijing, China; ^2^ Beijing Key Laboratory of Molecular Targeted Diagnosis and Therapy in Nuclear Medicine, Beijing, China; ^3^ Department of Endocrinology, State Key Laboratory of Complex Severe and Rare Diseases, Peking Union Medical College Hospital Chinese Academy of Medical Sciences and Peking Union Medical College, Beijing, China

**Keywords:** ^68^Ga-DOTA-TATE, ^68^Ga-DOTA-JR11, TIO, causative tumor, multiple

## Abstract

**Background:**

The purpose of this study is to compare the sensitivity of ^68^Ga-DOTA-JR11 and ^68^Ga-DOTA-TATE PET/CT for detecting the responsible tumor of tumor-induced osteomalacia (TIO) and investigate if ^68^Ga-DOTA-JR11 PET/CT can identify the culprit tumor of TIO in multiple suspicious lesions in ^68^Ga-DOTA-TATE PET/CT.

**Methods:**

A total of 19 patients with suspected TIO were prospectively recruited in this study. Each patient underwent whole-body PET/CT scan 40–60 min postinjection using ^68^Ga-DOTA-TATE and ^68^Ga-DOTA-JR11 on the same PET/CT, respectively in sequence, and on consecutive days. The diagnosis of TIO was confirmed by the combination of the postsurgical pathological results of the tumor and clinical information.

**Results:**

Among the 19 patients with TIO who were included in this study, culprit tumors from all patients were confirmed pathologically. ^68^Ga-DOTA-TATE PET/CT positively identified the causative tumor in 18/19 patients, whereas ^68^Ga-DOTA-JR11 PET/CT was positive in 11/19 patients (94.7% vs. 57.9%, respectively; *p* < 0.05). ^68^Ga-DOTA-TATE PET/CT demonstrated more than one increased focal activity in 7 patients for a total of 16 lesions (3 lesions each in 2 patients and 2 lesions each in the rest 5 patients). However, seven of these 16 lesions showed concordant results on ^68^Ga-DOTA-JR11 PET/CT by demonstrating increased activity (one lesion in each of the 7 patients). The surgical specimens of the lesions in these 7 patients confirmed the phosphaturic mesenchymal tumor. A total of 11 culprit tumors were positive in both ^68^Ga-DOTA-TATE and ^68^Ga-DOTA-JR11 PET/CT. The SUVmax of 11 culprit tumors was significantly higher on ^68^Ga-DOTA-TATE PET/CT compared with that on ^68^Ga-DOTA-JR11 PET/CT (17.8 ± 12.5 vs. 6.8 ± 6.2; *p* < 0.05).

**Conclusions:**

^68^Ga-DOTA-TATE PET/CT is more sensitive to ^68^Ga-DOTA-JR11 PET/CT in the detection of the culprit tumor of TIO. However, ^68^Ga-DOTA-JR11 PET/CT might be helpful to identify the tumor in multiple suspicious lesions in ^68^Ga-DOTA-TATE PET/CT.

**Clinical Trial Registration:**

clinicaltrials.gov, identifier NCT 04689893.

## Introduction

Tumor-induced osteomalacia (TIO), also known as oncogenic osteomalacia, is a rare paraneoplastic syndrome caused by excessive fibroblast growth factor 23 (FGF23) production by a tumor of mesenchymal origin (1, 2). The symptoms of TIO are progressive bone pain, muscle weakness, dyskinesia, bone deformation, and height loss (3). The key to the cure of TIO is surgical resection of the culprit tumor.

Somatostatin receptor (SSTR) imaging with PET has high specificity for the detection of the culprit tumors of TIOs, because they express high levels of SSTR, mainly subtype 2 (4). Over the last decade, ^68^Ga-labeled SSTR-based imaging has made a significant impact in detecting the culprit tumor of TIO (5–16). ^68^Ga-DOTA-TATE is the most widely used SSTR PET tracer for the detection of TIO, and it was recommended as a first-line imaging method for localization of the causative tumor (17).

However, false positivity, including fracture and/or inflammation, in ^68^Ga-DOTA-TATE PET/CT is a challenge in image interpretation, which may make the causative tumor indistinguishable in multiple suspicious lesions. To some extent, Ding Jie et al. (13) and Singh Deepa et al. (15) have come up with some solutions to avoid image misjudgment. Unfortunately, there is no effective way to identify multiple suspicious lesions with intensively increased uptake on ^68^Ga-DOTA-TATE PET/CT.

The significant development of SSTR antagonist tracers may make it possible to distinguish the multiple suspected lesions. JR11 (Cpa-c[d-Cys-Aph(Hor)-d-Aph(Cbm)-Lys-Thr-Cys]-d-Tyr-NH2) stands out from many SSTR subtype 2 antagonists due to its best comprehensive biological characteristics and may have a potential distribution advantage *in vivo* (18). Thus, ^68^Ga-DOTA-JR11 was developed as an SSTR2-specific antagonist for PET tracer. However, the SSTR2 affinity of ^68^Ga-DOTA-JR11 is lower than ^68^Ga-DOTA-TATE (19), which means that the uptake of ^68^Ga-DOTA-JR11 by suspected lesions may be lower than that of ^68^Ga-DOTA-TATE. The purpose of this prospective study is to compare the sensitivity of ^68^Ga-DOTA-JR11 and ^68^Ga-DOTA-TATE PET/CT for detecting the responsible tumor of TIO and investigate if ^68^Ga-DOTA-JR11 PET/CT can identify the culprit tumor of TIO in multiple suspicious lesions.

## Materials and Methods

### Study Design and Patient Population

This study was registered at clinicaltrials.gov (NCT 04689893 registered at 28/12/2020) and approved by the Institute Review Board of Peking Union Medical College Hospital (PUMCH) (IRB protocol #S-418). Statement of informed consent (The informed consent was obtained from parents for participants under 18 years of age.) was obtained from all patients recruited in the study. All patients had chronic disease with typical symptoms of TIO, unexplained hypophosphatemia, and increased FGF-23 levels and were referred to us by endocrinologists of PUMCH with a clinical diagnosis of TIO. The two PET/CT scans were conducted on two consecutive days. This research has been performed in accordance with the Declaration of Helsinki.

### ^68^Ga-DOTA-TATE and ^68^Ga-DOTA-JR11 Preparation and Imaging

^68^Ga-DOTATATE and ^68^Ga-DOTA-JR11 were produced following our previously published procedure (20). The study was carried out on a time-of-flight PET/CT scanner (Polestar m660, SinoUnion Healthcare Inc., Beijing, China) on two consecutive days. Patients received an intravenous injection of ^68^Ga-DOTATATE (111-148 MBq, 40 μg) on the first day and ^68^Ga-DOTA-JR11 (111-148 MBq, 40 μg) on the second day. A low-dose whole-body CT scan (120 keV; 100 mAs; 1.3 pitch; 2.5 mm slice thickness; 0.5 s rotation time; estimated radiation dose 9.0 mGy) was obtained at 40–60 min postinjection for anatomical localization and attenuation correction. PET scanning followed at 1.5 min/bed position with a 23-slice overlap. Images were reconstructed using an ordered subset expectation maximization algorithm (2 iterations, 10 subsets, 192 × 192 matrix) and corrected for CT-based attenuation, dead time, random events, and scatter.

### Image Interpretation

The images were interpreted jointly by 2 experienced nuclear medicine physicians who were not aware of clinical chart records. The uptake and anatomical changes of suspicious sites on ^68^Ga-DOTA-TATE and ^68^Ga-DOTA-JR11 PET/CT were recorded and analyzed separately. Conflicts in the two reports were resolved by rediscussing and finally reached a consensus. The diagnosis of TIO was confirmed by the combination of the postsurgical pathological results of the tumor, normalization of serum phosphate level after the excision of the culprit tumor, and symptomatic improvement. Any abnormal high uptake of ^68^Ga-DOTA-TATE and ^68^Ga-DOTA-JR11, significantly higher than that of the surrounding tissue, that cannot be explained by physiological activity is considered significantly high.

### Statistical Analysis

The sensitivity of ^68^Ga-DOTA-TATE and ^68^Ga-DOTA-JR11 PET/CT regardless of tumor sites was calculated on a per-patient basis in this study. All calculations were performed using SPSS (IBM SPSS Statistics for Windows, Version 21.0. Armonk, NY). The chi-squared test was used to statistically compare the difference between the different study groups. *p*-values < 0.05 were considered statistically significant.

## Results

**Figure 1:**
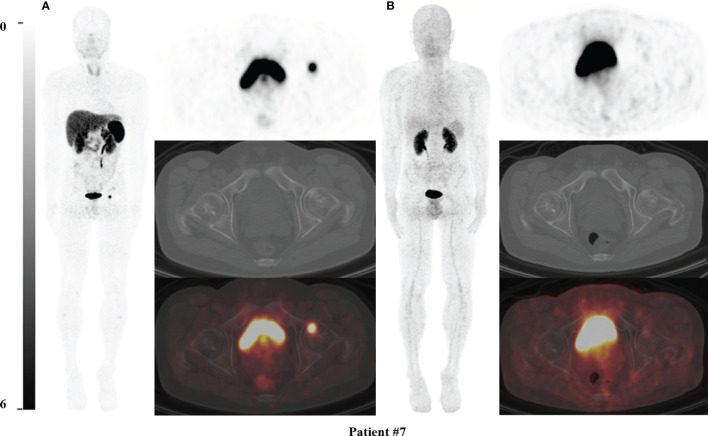
^68^Ga-DOTA-TATE PET/CT comparing with ^68^Ga-DOTA-JR11 PET/CT in Patient #7. The causative tumors of TIO were found in the left femoral head in patient #7 **(A)** on ^68^Ga-DOTA-TATE PET/CT, but negative on ^68^Ga-DOTA-JR11 PET/CT **(B)**. The lesion of the left femoral head was confirmed as the causative tumor of TIO by postsurgical pathological results.

19 patients (9 women, 10 men, aged 12–60 years [39.5 ± 13.8 years]]) with suspicious TIO were consecutively enrolled in the study from March 2020 to December 2020. The clinical characteristics are summarized in **Table 1**. Among the 19 patients with TIO who were included in this study, culprit tumors from all patients were confirmed pathologically. ^68^Ga-DOTA-TATE PET/CT positively identified the causative tumor in 18/19 patients, whereas ^68^Ga-DOTA-JR11 PET/CT was positive in 11/19 patients (94.7% vs. 57.9%, respectively; *p* < 0.05; **Figure 1**). Patient #14 was negative with both examinations. This lesion showed no increased uptake on PET but an expansive osteolytic disorder of mandible on CT, the lesion was subsequently resected, and the diagnosis of the culprit tumor of TIO was confirmed by the combination of the pathological results, normalization of serum phosphate level after the excision, and symptomatic improvement.

**Table 1:** ^68^Ga-DOTA-TATE and ^68^Ga-DOTA-JR11 PET/CT findings in patients with TIO.

No.	Age	Gender	^68^Ga-DOTA-TATE	^68^Ga-DOTA-JR11	The site of culprit tumor*^a^*
1	28	Male	A single positive lesion	Negative	Right tibia
2	28	Male	A single positive lesion	Negative	Left maxilla
3	47	Male	Multiple positive lesions (tongue, left femoral head)	A single positive lesion (tongue)	Soft tissue at the tongue
4	48	Male	A single positive lesion	Negative	Soft tissue of right lower limb
5	30	Female	Multiple positive lesions (left tibia, left fibula, soft tissue around the left knee joint)	A single positive lesion (left tibia)	Left tibia
6	34	Female	Multiple positive lesions (left femoral head, left pubic bone)	A single positive lesion (left femoral head)	Left femoral head
7	40	Male	A single positive lesion	Negative	Left femoral head
8	54	Female	Multiple positive lesions (left fibula, bilateral femur)	A single positive lesion (left fibula)	Left fibula
9	41	Male	Multiple positive lesions (left femoral condyle, right femoral neck)	A single positive lesion (left femoral condyle)	Left femoral condyle
10	12	Female	A single positive lesion (soft tissue behind the left knee joint)	A single positive lesion (soft tissue behind the left knee joint)	Soft tissue behind the left knee joint
11	55	Female	A single positive lesion (sacrum)	negative	Sacrum
12	26	Female	A single positive lesion (right humerus)	A single positive lesion (right humerus)	Right humerus
13	48	Female	Negative	Negative	Right mandible
14	53	Male	Multiple positive lesions (right second rib, left femoral neck)	A single positive lesion (right second rib)	Right second rib
15	49	Female	A single positive lesion (left maxilla)	A single positive lesion (left maxilla)	Left maxilla
16	18	Female	Multiple positive lesions (left femur, left ilium)	A single positive lesion (left femur)	Left femur
17	60	Male	A single positive lesion (left second rib)	A single positive lesion (left second rib)	Left second rib
18	28	Male	A single positive lesion (soft tissue of left lower limb)	Negative	Soft tissue of left lower limb
19	53	Male	A single positive lesion (left ilium)	Negative	Left ilium

^a^The site of culprit tumor was confirmed by pathology.

**Figure 2:**
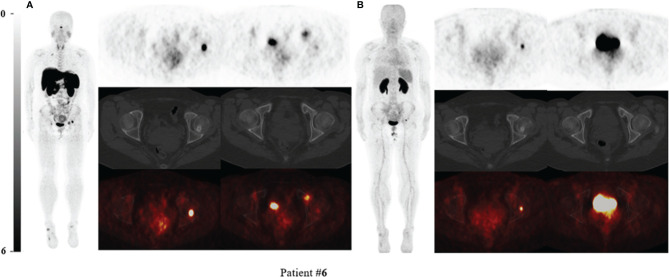
^68^Ga-DOTA-TATE PET/CT compared with ^68^Ga-DOTA-JR11 PET/CT in Patient #6 with multiple suspected lesions. Two intensive uptake lesions in the left femoral head and left pubic bone revealed on ^68^Ga-DOTA-TATE PET/CT **(A)**, which suggested that they might be the culprit tumors. The lesion of the left femoral head showed osteogenic change. However, the lesion in the left pubic bone only showed slightly increased uptake and the focus in the left femoral head still showed high uptake on ^68^Ga-DOTA-JR11 PET/CT **(B)**. The lesion of the left femoral head was confirmed as the responsible tumor of TIO by postsurgical pathological results.

^68^Ga-DOTA-TATE PET/CT demonstrated more than one increased focal activity in 7 patients for a total of 16 lesions (3 lesions each in 2 patients and 2 lesions each in the rest 5 patients). The sites of these 16 suspicious lesions were tongue (n = 1), left tibia (n = 1), left femoral head (n = 2), left fibula (n = 2), left femoral condyle (n = 1), right second rib (n = 1), femoral shaft (n = 3), femoral neck (n = 2), left ilium (n = 1), left pubic bone (n = 1), and soft tissue around the left knee joint (n = 1) (**Table 1**). Among the 14 lesions in the bone, 10 did not correspond to any morphology change on CT, one had typical fracture line (left fibula), 1 had lytic change, and 2 had sclerotic changes. Seven of these 16 lesions showed concordant results on ^68^Ga-DOTA-JR11 PET/CT by demonstrating increased activity (one lesion in each of the 7 patients; **Figure 2**). ^68^Ga-DOTA-JR11 PET/CT did not reveal other abnormal activity in these patients. The 7 lesions that were both ^68^Ga-DOTA-TATE and ^68^Ga-DOTA-JR11 positive were considered more likely to be the causative tumor of osteomalacia (the fibula lesion with the fracture line was one of them, Patient **#8**). The attempt of removing these lesions was first made. The surgical specimens of the lesions in these 7 patients confirmed the phosphaturic mesenchymal tumor, indicating that the 7 lesions were true positive on ^68^Ga-DOTA-TATE PET/CT and ^68^Ga-DOTA-JR11 PET/CT. More importantly, the patients’ symptoms promptly improved and the postsurgical serum phosphate level returned to normal after surgery. This finding indicated that the other 9 lesions seen on ^68^Ga-DOTA-TATE PET/CT were not the cause of osteomalacia.

A total of 11 culprit tumors were positive in both ^68^Ga-DOTA-TATE and ^68^Ga-DOTA-JR11 PET/CT. The SUVmax of 11 culprit tumors was significantly higher on ^68^Ga-DOTA-TATE PET/CT compared with that on ^68^Ga-DOTA-JR11 PET/CT [17.8 ± 12.5; median, 11.4; (range: 7.0–47.7) vs. 6.8 ± 6.2; median, 4.49; (range: 1.7–21.5), *P*<0.05] (**Table 2**).

**Table 2:** Clinical features of TIO patients, including histopathological characteristics and SUVmax of the responsible tumor of TIO.

Patient no.	Age	Gender	^68^Ga-DOTA-TATE	^68^Ga-DOTA-JR11	Histopathological characteristics
3	47	Male	47.7	14.5	Phosphaturic mesenchymal tumor
5	30	Female	19.4	9.1	Phosphaturic mesenchymal tumor
6	34	Female	26.4	7.3	Phosphaturic mesenchymal tumor
8	54	Female	30.5	21.5	Phosphaturic mesenchymal tumor
9	41	Male	11.0	4.5	Phosphaturic mesenchymal tumor
10	12	Female	7.1	2.1	Phosphaturic mesenchymal tumor
12	26	Female	14.9	2.9	Phosphaturic mesenchymal tumor
14	53	Male	11.4	1.7	Phosphaturic mesenchymal tumor
15	49	Female	9.5	3.6	Phosphaturic mesenchymal tumor
16	18	Female	7.0	1.8	Phosphaturic mesenchymal tumor
17	60	Male	10.9	6.3	Phosphaturic mesenchymal tumor
			*p* < 0.05		

## Discussion

^68^Ga-DOTA-TATE PET/CT was recommended as the first-line imaging investigation depending on high sensitivity (about 90%) in TIO lesion localization (17, 21). However, multiple non-causative lesions with intensive activity were revealed on ^68^Ga-DOTA-TATE PET/CT sometimes, which might lead to a failure for localization of the culprit tumor due to fracture sites in osteoporotic bones and/or inflammation. Ding et al. (13) reported that the mild activity at the sites of fracture is not challenged in the interpretation of ^68^Ga-DOTA-TATE PET/CT in the patients with TIO. However, their study did not discuss how to distinguish between non-specific high uptake and tumors, which is the real challenge of image interpretation. In the present study, by combining ^68^Ga-DOTA-TATE and ^68^Ga-DOTA-JR11 PET/CT, we successfully identified 7 true TIO lesions in 16 suspected lesions in 7 patients. The lower uptake of ^68^Ga-DOTA-JR11 than ^68^Ga-DOTA-TATE has been explained by Zhu et al. (20). The main reason is that ^68^Ga-DOTA-TATE enjoys 100 times higher affinity for SSTR2 than ^68^Ga-DOTA-JR11, rendering a worse tumor uptake of ^68^Ga-DOTA-JR11 (18, 22).

Deepa et al.’s study (15) demonstrated that the SUVmax of fracture tends to be lower than that of tumor on ^68^Ga-DOTA-NOC PET/CT, but overlap lies between them, leading to misjudgment of the results of some TIO patients. The authors also mentioned that the culprit tumor of TIO has some related soft tissue components, but the tumor is generally small, which makes this interpretation difficult to achieve. In addition, the tumor sometimes has no morphological abnormality on CT (13). In this study, the uptake of 9 non-specific intensive activities on ^68^Ga-DOTA-TATE PET/CT reduced significantly to the background level on ^68^Ga-DOTA-JR11 PET/CT. Although the uptake of the 7 causative tumors is also reduced on ^68^Ga-DOTA-JR11 PET/CT, the increase in uptake of the tumor remains to be significantly observed because of the high tumor-to-background ratio. This may be due to the fact that the expression of SSTR2 in causative tumors of TIO is higher than that in non-specific uptake tissues, including fracture and inflammation. The results of this study may be of great significance for the accurate localization of tumors in patients with TIO as accurate location-guided surgical resection is the key to treatment and recovery (1). In an algorithm of locating the tumor of TIO, it was reported that venous sampling is particularly useful to confirm causative tumors in patients with multiple suspicious regions (1). However, such clinical test requires high technical skills to operate and rich prior knowledge to interpret results. This prospective study shows the feasibility and effectiveness of ^68^Ga-DOTA-JR11 PET/CT in identifying multiple suspected lesions with high specificity.

Compared with ^68^Ga-DOTA-TATE PET/CT, ^68^Ga-DOTA-JR11 PET/CT shows an inferior detection ability for the causative tumor of TIO. The reason for the difference due to their different affinity for SSTR2 has been explained above. In this study, most of the tumors of TIO were located in bone. The sensitivity of ^68^Ga-DOTA-JR11 in detecting bone tumors was lower than that of ^68^Ga-DOTA-TATE, and the SUVmax of lesions on ^68^Ga-DOTA-JR11 was lower than that of ^68^Ga-DOTA-TATE.

The limitation of this study is that the number of TIO patients with multiple suspicious lesions is small, and more data will be needed in the future.

In conclusion, in the culprit tumor of TIO detection, ^68^Ga-DOTA-TATE PET/CT is more sensitive than ^68^Ga-DOTA-JR11 PET/CT (detecting 18/19 vs. 11/19 lesions) but less specific as it shows more false positives. ^68^Ga-DOTA-JR11 PET/CT might be helpful to identify the tumor of multiple suspicious lesions in ^68^Ga-DOTA-TATE PET/CT.

## Data Availability Statement

The original contributions presented in the study are included in the article/supplementary material. Further inquiries can be directed to the corresponding authors.

## Ethics Statement

This study was registered at clinicaltrials.gov (NCT 04689893 registered at 28/12/2020) and approved by the Institute Review Board of Peking Union Medical College Hospital (PUMCH) (IRB protocol #S-418). Written informed consent to participate in this study was provided by the participants’ legal guardian/next of kin. Written informed consent was obtained from the individual(s), and minor(s)’ legal guardian/next of kin, for the publication of any potentially identifiable images or data included in this article.

## Author Contributions

GH, YZ, YL, PW, LH, WX, XX, FL, and HJ contributed to the design and implementation of the research, to the analysis of the results, and to the writing of the manuscript. All authors contributed to the article and approved the submitted version.

## Conflict of Interest

All authors declare that the research was conducted in the absence of any commercial or financial relationships that could be construed as a potential conflict of interest.

## Publisher’s Note

All claims expressed in this article are solely those of the authors and do not necessarily represent those of their affiliated organizations, or those of the publisher, the editors and the reviewers. Any product that may be evaluated in this article, or claim that may be made by its manufacturer, is not guaranteed or endorsed by the publisher.
